# Preeclampsia Is Associated with Alterations in the p53-Pathway in Villous Trophoblast

**DOI:** 10.1371/journal.pone.0087621

**Published:** 2014-01-30

**Authors:** Andrew N. Sharp, Alexander E. P. Heazell, Dora Baczyk, Caroline E. Dunk, Helen A. Lacey, Carolyn J. P. Jones, Jonathan E. Perkins, John C. P. Kingdom, Philip N. Baker, Ian P. Crocker

**Affiliations:** 1 Maternal & Fetal Health Research Centre, Manchester Academic Health Science Centre, University of Manchester, Manchester, United Kingdom; 2 Samuel Lunenfeld Research Institute, Mount Sinai Hospital, Toronto, Canada; 3 Liggins Institute, University of Auckland, Auckland, New Zealand; Université de Montréal, Canada

## Abstract

**Background:**

Preeclampsia (PE) is characterized by exaggerated apoptosis of the villous trophoblast of placental villi. Since p53 is a critical regulator of apoptosis we hypothesized that excessive apoptosis in PE is mediated by abnormal expression of proteins participating in the p53 pathway and that modulation of the p53 pathway alters trophoblast apoptosis *in vitro*.

**Methods:**

Fresh placental villous tissue was collected from normal pregnancies and pregnancies complicated by PE; Western blotting and real-time PCR were performed on tissue lysate for protein and mRNA expression of p53 and downstream effector proteins, p21, Bax and caspases 3 and 8. To further assess the ability of p53 to modulate apoptosis within trophoblast, BeWo cells and placental villous tissue were exposed to the p53-activator, Nutlin-3, alone or in combination with the p53-inhibitor, Pifithrin-α (PFT- α). Equally, Mdm2 was knocked-down with siRNA.

**Results:**

Protein expression of p53, p21 and Bax was significantly increased in pregnancies complicated by PE. Conversely, Mdm2 protein levels were significantly depleted in PE; immunohistochemistry showed these changes to be confined to trophoblast. Reduction in the negative feedback of p53 by Mdm2, using siRNA and Nutlin-3, caused an imbalance between p53 and Mdm2 that triggered apoptosis in term villous explants. In the case of Nutlin, this was attenuated by Pifithrin-α.

**Conclusions:**

These data illustrate the potential for an imbalance in p53 and Mdm2 expression to promote excessive apoptosis in villous trophoblast. The upstream regulation of p53 and Mdm2, with regard to exaggerated apoptosis and autophagy in PE, merits further investigation.

## Introduction

Preeclampsia (PE), defined as new onset hypertension and proteinuria, is a severe multi-system disorder affecting 3–6% of human pregnancies [1], [2]. The aetiology of PE is unclear, but when manifest from mid-gestation it is often associated with significant placental pathology. The underlying placental phenotype is complex and ill-defined. However, there is profound cellular dysfunction with increased unfolded protein response, apoptosis, autophagy and necrosis of villous trophoblast [3], [4], [5]. These processes may promote the release of factors from the placenta which trigger maternal endothelial activation and/or systemic inflammation [6], [7].

As a source of such factors, the multinucleated syncytiotrophoblast of the placenta is in direct contact with maternal blood [6]. It is generated by mitosis and fusion from underlying villous cytotrophoblasts. Over time syncytial nuclei gather in structures, more recently termed syncytial nuclear aggregates (SNAs) [8] but recently it has been observed that apoptosis may not be a significant mechanism in this decline [9]. In the human placenta, evidence of trophoblast apoptosis increases with advancing gestation [10], whilst proliferating cytotrophoblasts become increasingly dispersed. In severe PE, cell turnover is dysregulated, resulting in decreased syncytiotrophoblast area [11], [12], increased apoptosis [4], [13], [14], autophagy [3] and increased density of SNAs [15], [16]. The role that this exaggerated apoptosis plays in placental pathology in PE is unclear, but ultimately it may prevent replenishment of syncytiotrophoblast, promote syncytial degeneration and release vasoactive or inflammatory factors into the maternal circulation [17].

The precise intracellular mechanisms that promote exaggerated apoptosis in PE are unknown, though many constituents are present [18]. In PE, apoptosis is thought to result from intrinsic cell damage, hypothesized *in vivo* to result from hypoxia-reperfusion injury [19], and replicated *in vitro* by hypoxia [20], [21], [22] and oxidative stress [23], [24].

A key regulatory component of apoptosis, resulting from intrinsic damage, is p53, a tumor suppressor responsible for maintaining genomic stability and tissue homeostasis [25]. In response to noxious stimuli, p53 promotes the downstream transcription of elements involved in apoptosis and cell-cycle arrest, including p21*^WAF-1^*, a cyclin-dependent kinase inhibitor [26], [27], APAF-1, an apoptosome component accountable for caspase activation [28], Puma, a potent inducer of apoptosis [29], and Bax, which increases mitochondrial membrane permeability, releasing factors further promoting caspase activation [26], [28].

Under normal circumstances cellular p53 is present at low concentrations, restrained by Mdm2 [30] which removes it from the nucleus and targets it for ubiquitination via the proteosome [31]. p53 promotes Mdm2 transcription creating an important negative feedback loop, where the balance between p53 and Mdm2 is essential for cell survival, as demonstrated by the embryonic lethality of mdm2 knockout in mice, although rescued by concomitant p53 knockout [32].

A role for p53 in exaggerated trophoblast apoptosis in PE is hypothesized, as p53 expression is increased in fetal growth restriction (FGR) - a related placental condition [22], [33]. In this study, we consider whether increased apoptosis in PE is associated with altered p53 expression and its downstream proteins. We have investigated the effects of its deregulation using two approaches: i) RNA interference to reduce p53 and Mdm2 expression in normal term villous trophoblast, and ii) pharmacological modulation of the p53 pathway, using Nutlin-3 and Pifithrin-α. Nutlin-3 competitively occupies the p53-Mdm2 binding cleft, stabilising p53 and increasing target gene transcription [34], [35], [36] and thus apoptosis [37], [38]. Pifithrin-α is a specific reversible inhibitor of p53 [39], capable of inhibiting p53-dependent gene transactivation [39], [40].

## Methods

Unless otherwise stated, all reagents were purchased from Sigma Chemical Co., Poole, UK.

### Tissue collection and preparation

A North-West Research Ethics Committee (08/H1010/55) and the research ethics board of Mount Sinai Hospital (Toronto, Canada) gave approval for this study. All participants gave written informed consent. Placentas were obtained from women with PE after 28 weeks gestation (n = 8) or from uncomplicated pregnancies (n = 8), within 30 minutes of delivery. PE was defined as a blood pressure of >140/90 mmHg on two or more occasions after the 20th week of pregnancy in a previously normotensive woman in the presence of significant proteinuria (either >300 mg/l in a 24 hour period or >2+ on a voided urine sample in the absence of urinary tract infection) [41]. Women with chronic hypertension, underlying renal disease, and/or insulin-dependent diabetes were excluded, as were pregnancies with co-existent FGR (<10^th^ centile individualised birthweight) [42].

Five areas of each placenta were sampled randomly from midway between the chorionic and basal plates. Collected fresh tissue was homogenised immediately on ice in lysis buffer (10 mM Hepes, 250 mM sucrose, 1 mM EDTA and protease inhibitor cocktail, pH 7.4) and homogenates were centrifuged at x9500 g for 3 minutes and supernatant collected, or fixed in 4% normal saline formalin (pH 7.2) prior to wax embedding.

### Preparation of pharmacological agents

Nutlin-3 and Pifithrin-α were dissolved in dimethyl sulphoxide (DMSO) to generate 1 mM stock solutions. Experimental media containing Nutlin-3 or Pifithrin-α were created by dilution in Dulbecco's modified Eagle's medium (DMEM)-F12 media supplemented with antibiotics (30 mg/l penicillin, 50 mg/l streptomycin and 150 µg/l glutamine) and 10% (v/v) Fetal Bovine Serum (FBS). Additional DMSO was added to all experimental wells to equalize concentrations (1.1% (v/v)). For each experiment, cells were exposed to DMSO vehicle controls, Nutlin-3 alone (30 µM) or Nutlin-3 (30 µM) combined with Pifithrin-α (10 µM). Concentrations were chosen from previous investigations (data not shown).

### BeWo cell cultures

BeWo cells were obtained from The European Collection of Animal Cell Cultures (Porton Down, Wiltshire, UK). Cells were grown to confluence under standard culture conditions in DMEM-F12 supplemented with antibiotics (same above) and 10% (v/v) FBS. Once confluent, cells were re-seeded in 96 well plates (2×10^4^ cells/well) (Corning Inc., NY, USA). All experiments were performed between passages 3 to 10.

To generate protein, BeWo cells were cultured in 25 cm^2^ flasks (Corning) until 80% confluent. Cells were then exposed to 30 µM Nutlin-3, 30 µM Nutlin-3 with 10 µM Pifithrin-α, or vehicle control alone for 24 hours (n = 5). The media was subsequently discarded and cells washed in ice cold phosphate buffered saline (PBS), before being scraped into HEPES buffer (25 mM HEPES, 5 mM MgCl_2_, 5 mM EDTA, 5 mM DTT, 2 mM PMSF and proteinase inhibitors). The solution was centrifuged at 400 g for 5 minutes and the resultant supernatant stored at −20°C.

### Culture and Transfection of Placental Villous Explants

For Nutlin and Pifithrin-α experiments, placental tissue was collected from uncomplicated pregnancies (n = 5) and cultured as previously described [20]. For transfection with siRNA (n = 6), three areas of each placenta were randomly sampled; villous tissue was dissected into single villous explants (weighing approximately 10 mg each), as previously described [43], [44]. These were mounted in polystyrene cubes and were allowed to float in DMEM supplemented with 1% (v/v) insulin, transferrin and selenium (ITS) at 37°C in 8% oxygen (placental normoxia). After 24 hours, culture medium was refreshed with medium containing the relevant siRNA sequence. Previously published siRNA oligonucleotides to p53 (Sense - GCAUGAACCGGAGGCCCAU, Antisense – AUGGGCCUCCGGUUCAUGC) [45] and Mdm2 (Sense – AAGGAAUAAGCCCUGCCCA, Antisense – UGGGCAGGGCUUAUUCCUU) [46] were used. Sequence homology was confirmed by BLAST search. Control experiments with no siRNA and scrambled siRNA sequence were included (Sense –UUCUCCGAACGUGUCACG; Antisense – ACGUGACACGUUCGGAGA). Explants were cultured for a further 48 hours, then villous tissue fixed in 4% (v/v) saline formalin (pH 7.2), prior to wax embedding. Alternatively, 2% (v/v) glutaraldehyde was used prior to electron microscopy. Culture medium was collected and stored (20°C) from each well.

### Quantitative PCR

Total RNA was extracted from fresh placental tissue and explants, and quantified as previously described [47]. 100 ng RNA was used for each batch of reverse transcription (RT). For experiments involving placental tissue, a calibrator sample of quantitative reference RNA (Stratagene) was included. For each gene, all samples were included in a single run. cDNA synthesis in the absence of RT enzyme were included as a negative control. cDNA synthesis was performed as previously described [48]. The specific primers were: p53 Forward (*F*) CTCCTCAGCATCTTATCCGAGTG, Reverse (*R*) GTGGTACAGTCAGAGCCAACC, Mdm2 – *F* GTGAAGGAAACTGGGGAGTCTT *R* AGGTACAGACATTTTGGTATT GCA, p21 – *F* AGGTGGACCTGGAGACTCTCA, *R* CGGCGTTTGGAGTGGTAGAAA, Bax – *F* GCTGTTGGGCTGGATCCAAG, *R* TCAGCCCATCTTCCAGA, Puma *F* AAAACTCACCAAACCAGAGCA, *R* GCTTTCCATTCCGTTTCTTTT, SDHA *FTGGGAACAAGAGGGCATCTG, RCCACCACTGCATCAAATTCATG* [20]. mRNA expression was quantified by SYBR Green I. Forty cycles of PCR were performed in triplicate: initial enzyme activation and template denaturation for 10 min at 95°C, followed by 30 s at 95°C, 1 min annealing at 55–59°C and an extension phase for 30 s at 72°C. At the end of the PCR run, dissociation curves were performed to ensure a single amplicon. In these experiments Rn was equal to the fluorescence emission intensity of the reporter dye normalized to ROX and dRn (the Rn of an unreacted sample minus the Rn value of the reaction). For each gene transcript, an amplification plot was created from dRn vs. product cycle threshold (Ct). Ct values of placental tissue were used to calculate a standard curve, constructed from the cDNA of the human reference RNA. For placental tissues, samples were normalized to the calibrator (cDNA from human reference RNA). For isolated cells, normalization was performed against Succinate dehydrogenase complex, subunit A (SDHA). All assays were between 93 and 105% efficient.

### Protein Preparation and Immunoblotting

Protein content was determined using a standardized commercial assay (Bio-Rad Laboratories Ltd, Hemel Hempstead, UK). 40 µg of tissue lysate were subjected to 10% discontinuous SDS-PAGE and transferred to a PVDF membrane.

Membranes were blocked for 1 hr with 3% (w/v) milk in Tris-buffered saline containing 0.05% (v/v) Tween-20 (TBS-T) and then probed overnight at 4°C, with mouse monoclonal antibodies to either p53 (Clone D01, Merck Biosciences, Nottingham, UK, 1 µg/ml (Explants), 0.1 µg/ml (BeWo)), Mdm2 (Clone 2A10, Merck Biosciences, 2 µg/ml), anti-p21 (Clone EA10, Merck Biosciences, 1∶100), Bak (TC-102, Merck Biosciences, 1∶200), Bcl-2 (Clone 100/D5, Abcam, Cambridge, UK, 1 µg/ml), Procaspase-3 (Clone 84803, R&D Systems, Abingdon, UK, 1∶1000), Procaspase 8 (Clone 84131, Merck Biosciences, 1∶100), Myosin Light Chain (Clone MY21, Abcam, 0.1 µg/ml), β-actin, (Clone AC15, Sigma, 1∶10,000) or rabbit polyclonal antibody against Bax (ab7977, Abcam, 1 µg/ml (Explants), 0.2 µg/ml (BeWo)), p21 (Abcam, 0.2 µg/ml (BeWo)), Puma (Abcam, 4 µg/ml) or β-actin (Clone AC15, Sigma, 1∶10,000). Membranes were incubated subsequently with horseradish peroxidase-conjugated goat anti-mouse IgG or goat anti-rabbit IgG (Dako, Ely, UK 1∶1000) for 1 hr at room temperature. Resulting bands were visualized on photo-sensitive film (Amersham Biosciences Ltd, Chalfont St. Giles, UK) using enhanced chemiluminescence reagents (Pierce, Rockford, IL, USA). Densitometry was performed as previously described [20]; bands of interest were standardized against constitutively expressed proteins (β-actin, Myosin Light Chain), the levels of which are not disrupted in PE [20], [33].

### Caspase Activity Assays on Fresh Tissue

After removing the media and washing with PBS, eight explants for each experimental time and condition were homogenised with 200 µl ice-cold homogenisation buffer (10 mM HEPES, 2 mM EDTA, 0.1% CHAPS, 5 mM DTT, 10 µg/ml pepstatin A, 20 µg/ml leupeptin, 10 µg/ml aprotinin, 10% glycerol, pH 7.0). Following centrifugation, total protein of the supernatant was recorded and 40 µl homogenate added to 180 µl of reaction buffer (100 mM HEPES, 0.5 mM EDTA, 10% glycerol, 5 mM DTT, 50 µM Cytochrome c, 10 mM dATP, pH 7.0) containing either the caspase substrate (50 µM Ac-DEVD-amc (Calbiochem-Novabiochem UK Ltd, Nottingham, UK) and/or a broad spectrum caspase inhibitor (25 µM Z-Vad-FMK; Calbiochem-Novabiochem UK Ltd) [49]. The samples were transferred into wells of a 96 well black-walled flat-bottomed culture plate and incubated for 30 minutes at 37°C in the dark. The reaction was stopped with 100 µl 1% (v/v) sodium acetate, 175 mM acetic acid, and fluorescence measured at 380/460 nm with a FL500 plate reading fluorimeter (Bio-Tek Instruments, Inc., Vermont, USA). Enzyme activity was expressed as fluorescence units per mg protein.

### Immunohistochemistry

5 µm sections were cut and transferred to 3-aminopropyltriethoxysilane (APES) coated slides. Slides were deparaffinized and exposed to microwave pre-treatment with 10 mM citrate buffer, pH 6.0. Slides were further treated with 3% (v/v) hydrogen peroxide in methanol for 45 mins. Non-specific binding was blocked by exposure for 1 hr at room temperature with normal rabbit or goat serum (10% (v/v) in PBS). Sections were exposed to mouse monoclonal antibodies against p53 (Clone DO-7, Dako, 7.8 µg/ml), Mdm2 (Merck Biosciences, 2 µg/ml), p21 (Abcam, 1.6 µg/ml), Bak (Merck, 2 µg/ml), Bcl-2 (Abcam, 1 µg/ml), M30 (Roche, 0.165 µg/ml) or rabbit polyclonal antibody to Bax (Abcam, 2 µg/ml) overnight at 4°C. Matched concentrations of non-specific mouse immunoglobulin were used as negative controls. Slides were probed with biotin conjugated goat anti-mouse or anti-rabbit antibodies (Dako, 1∶200) for 1 hr at room temperature, followed by incubation with avidin-peroxidase (5 µg/ml in 0.125 M TBS+0.347 M NaCl [48]) for 30 mins at room temperature. Immunostaining was revealed by exposure to concentrated 3,3-diaminobenzidine for 3 mins. Slides were counterstained with methyl green or Harris' Haematoxylin and sections viewed by Leitz microscope with ImageProPlus 3.0 imaging software (Media Cybernetics Inc, Silver Spring, MD, USA). Immunoreactivity was undetectable in all negative controls.

### Assessment of Apoptosis and Syncytial Degeneration

Apoptosis was assessed by commercial TUNEL kit (Roche Applied Diagnostics, Sussex, UK), with modifications from the manufacturers' instructions as previously described [20]. For tissue studies, TUNEL staining was visualised by light microscopy of 5 randomly selected fields of terminal villi for each experimental condition. The number of TUNEL positive nuclei and total number of nuclei were assessed using a sequential colour thresholding technique using ImageProPlus 4.5 (Media Cybernetics Inc, Silver Spring, MD, USA) in which nuclei were first defined and then counted [12]. Apoptosis was also assessed with the M30 neo-epitope to cytokeratin-M30 which is a specific marker of caspase 3 activity in the trophoblast [50].

Apoptosis in BeWo cells was also assessed by TUNEL, after culture on glass coverslips. Cells were exposed to experimental conditions for 24 hrs prior to washing with PBS and then fixed with 4% (v/v) paraformaldehyde before stored at 4°C. Cells were subsequently washed, permeabilised with 0.1% (v/v) Triton X-100 and incubated with 100 µl TUNEL reagent (Roche Applied Diagnostics, Sussex, UK) per slide at 37°C for 1 hour. Coverslips were mounted on slides in Vector hardshield with DAPI (4′, 6-diamidino-2-phenylindole) (Vector laboratories, Peterborough, UK) and stored in the dark at 4°C prior to analysis. An Olympus IX70 fluorescent microscope (Olympus UK, Essex, UK) and QICAM FAST 1394 digital camera (QIMAGING Corporate, Surrey, Canada) were used. 10 images per field were taken per slide, for analysis with Qcapture Pro 6.0 (Media Cybernetics, Silver Spring, MD, USA). The number of TUNEL positive nuclei was recorded as a proportion of total DAPI positive cells (TUNEL positive index). Due to the uncertainty regarding the activity of nuclei within the different trophoblast compartments, no differentiation was made between cytotrophoblast and syncytiotrophoblast nuclei [51].

Cellular apoptosis was further quantified using Caspase-Glo® 3/7 kit (Promega, Madison, WI, USA), in accordance with the manufacturer's instructions. Cells were originally seeded in sterile 96-well white walled plates (2×10^4^/well) before pharmacological exposure. An equal volume of Caspase-Glo® 3/7 was subsequently added, with incubation in the dark for 1 hr at room temperature. Luminescence was the assessed using a GeminiXS (Molecular Devices, Sunnyvale, CA, USA). 1 µM staurosporine, a well characterized apoptosis inducer, was used as a positive control.

### Syncytial Nuclear Aggregate (SNA) Assessment

SNAs were assessed on 5 µm sections stained with haematoxylin and eosin. Ten randomly selected high-powered fields of terminal villi (magnification 400×) were considered for each experimental condition. To avoid bias, the microscope was taken out of focus between frames. An SNA was defined as an aggregate of 10 syncytiotrophoblast nuclei protruding from the villous surface, not in direct contact with adjacent villi. The number of SNAs was counted manually and trophoblast area measured by sequential colour thresholding as previously described [16]. Data were normalized to villous area to give a measure of SNAs per mm^2^.

### Assessment of Cytotrophoblast Proliferation

Cytotrophoblast proliferation was quantified using Ki-67 (Mib-1) immunostaining which has been used previously to determine proliferative activity within trophoblast [20], [52]. Tissue was prepared as for immunohistochemistry using mouse monoclonal anti-Ki-67 antibody (Dako, 1∶50). Five randomly selected areas of each section were assessed using light microscopy, positive nuclei were counted manually and total nuclei per villus defined, deriving a ratio of proliferative nuclei:total nuclei as previously described [20].

### Electron microscopy

Explants from each experimental condition (n = 3) were fixed and processed for electron microscopy, as previously described [53]. Semi-thin sections, 0.5 µm thick, were cut and stained with 1% (w/v) Toludine blue in 1% (w/v) borax. After inspection to identify areas of interest, ultrathin sections were cut using a diamond knife, mounted on copper grids and stained with uranyl acetate and lead citrate. These were examined using a Philips CM10 electron microscope at an accelerating voltage of 80 kV, and representative areas photographed.

### Statistical Analysis

Unless otherwise stated, statistical significance was tested using either Wilcoxon signed-rank test, Mann-Whitney U-test or Friedman test for non-parametric data, and results presented as medians and ranges; p≤0.05 were considered statistically significant. Data were analysed using GraphPad Prism (v4.0; San Diego, CA, USA) and represented as box and whisker plots.

## Results

### Study participants

The median values and ranges of participant demographics are given in Table 1. The PE pregnancies and controls showed no significant differences in gestational age, parity or mode of delivery. The mean arterial blood pressure was significantly elevated at sampling in the PE group.

**Table 1:** Demographic data for women with normal pregnancies and pre-eclampsia (PE).

	Normal (N = 8)	PE (N = 8)	Significance
Age (years)	26 (20 – 35)	31 (25–38)	NS
BMI	25 (19–41)	27 (21–33)	NS
Gravidity	2 (1–3)	2 (1–4)	NS
Parity	1 (0–2)	1 (0–2)	NS
MABP in First Trimester (mmHg)	82 (73–87)	88 (80–110)	NS
Maximum MABP (mmHg)	87 (81–95)	125 (113–141)	p <0.01
Gestation at delivery (weeks)	39^+3^ (37^+0^–41^+5^)	37^+0^(28^+3^–41^+3^)	NS
Mode of delivery	4 VD, 4 CS	4 VD, 4 CS	NS

MABP =  Mean Arterial Blood Pressure, VD =  Vaginal Delivery, CS =  Caesarean section.

### Expression of constituents of the p53 pathway and downstream effectors of apoptosis

**Figure 1:**
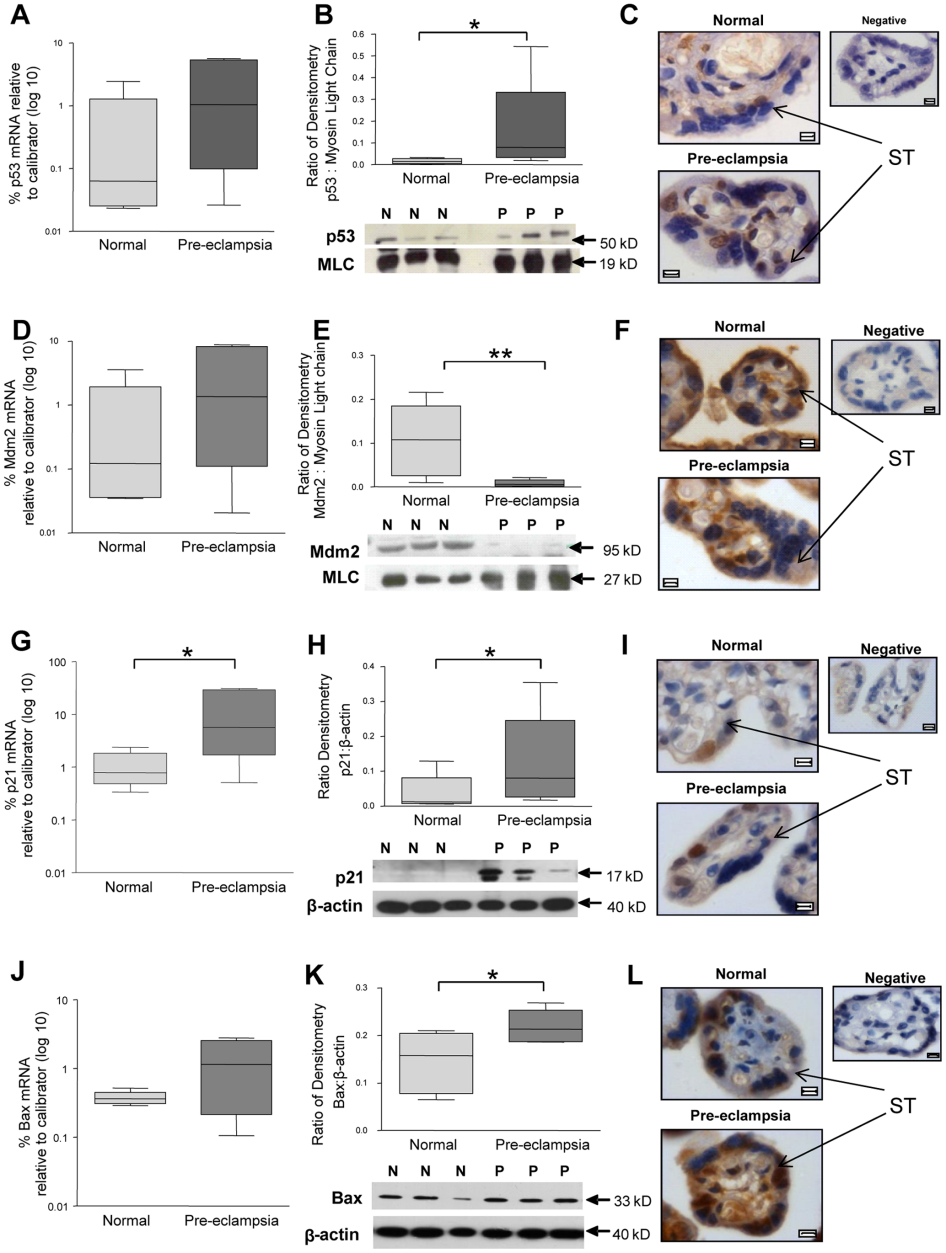
The expression of p53-pathway components in placentas of normal pregnancies and pregnancies complicated by pre-eclampsia (PE). (**A, D, G and J**) mRNA expression of p53, Mdm2, p21 and Bax respectively, in placental lysates at delivery. (**B, E, H, and K**) Protein expression of p53, Mdm2, p21 and Bax, respectively, through densitometry standardized to Myosin Light Chain (MLC) (*p<0.05, **p<0.01, n = 8). (**C, F, I, and L**) Localisation of p53, Mdm2, p21 and Bax respectively, in placental villi by immunohistochemistry. ST =  Syncytiotrophoblast. Images counterstained with haematoxylin. Scale bar  = 5 µm.

p53 mRNA levels were not significantly increased in pregnancies complicated by PE (Figure 1A). At the protein level, p53 was significantly elevated in placentas from pre-eclamptic pregnancies, (Figure 1B). In normal pregnancies, p53 was evident in occasional syncytiotrophoblast nuclei and rarely seen in cytotrophoblasts and syncytiotrophoblast cytoplasm (Figure 1C). In PE p53 localized to trophoblast nuclei in CT and ST compartments (Figure 1C). Staining was also noted in discrete areas of the syncytiotrophoblast cytoplasm.

In contrast, protein expression levels of Mdm2 were significantly reduced in PE although again there was no significant difference in mRNA levels (Figure 1D and 1E). Mdm2 was expressed in normal pregnancies throughout the syncytiotrophoblast cytoplasm (Figure 1F), and in the cytoplasm of some cytotrophoblasts and stromal cells (Figure 1F). In PE, Mdm2 immunostaining appeared to be decreased throughout the syncytiotrophoblast cytoplasm (Figure 1F).

The increased levels of p53 were associated with increased expression of downstream elements of the apoptotic pathway. p21 mRNA and protein levels were increased in PE (Figure 1G and H). In some cases of PE, p21 expression showed a double band consistent with cleavage by caspases. In normal pregnancy, p21 expression was confined to cytotrophoblast and syncytiotrophoblast nuclei, with no cytoplasmic expression in either cell type (Figure 1I). In PE, p21 expression appeared to be localized to the trophoblast layer (Figure 1I). Bax protein was significantly increased in the PE cases but Bax mRNA was not significantly elevated (Figure 1J and K). Bax was expressed throughout the syncytiotrophoblast, cytotrophoblast and occasional stromal cytoplasm in normal and PE placental tissue (Figure 1L).

**Figure 2:**
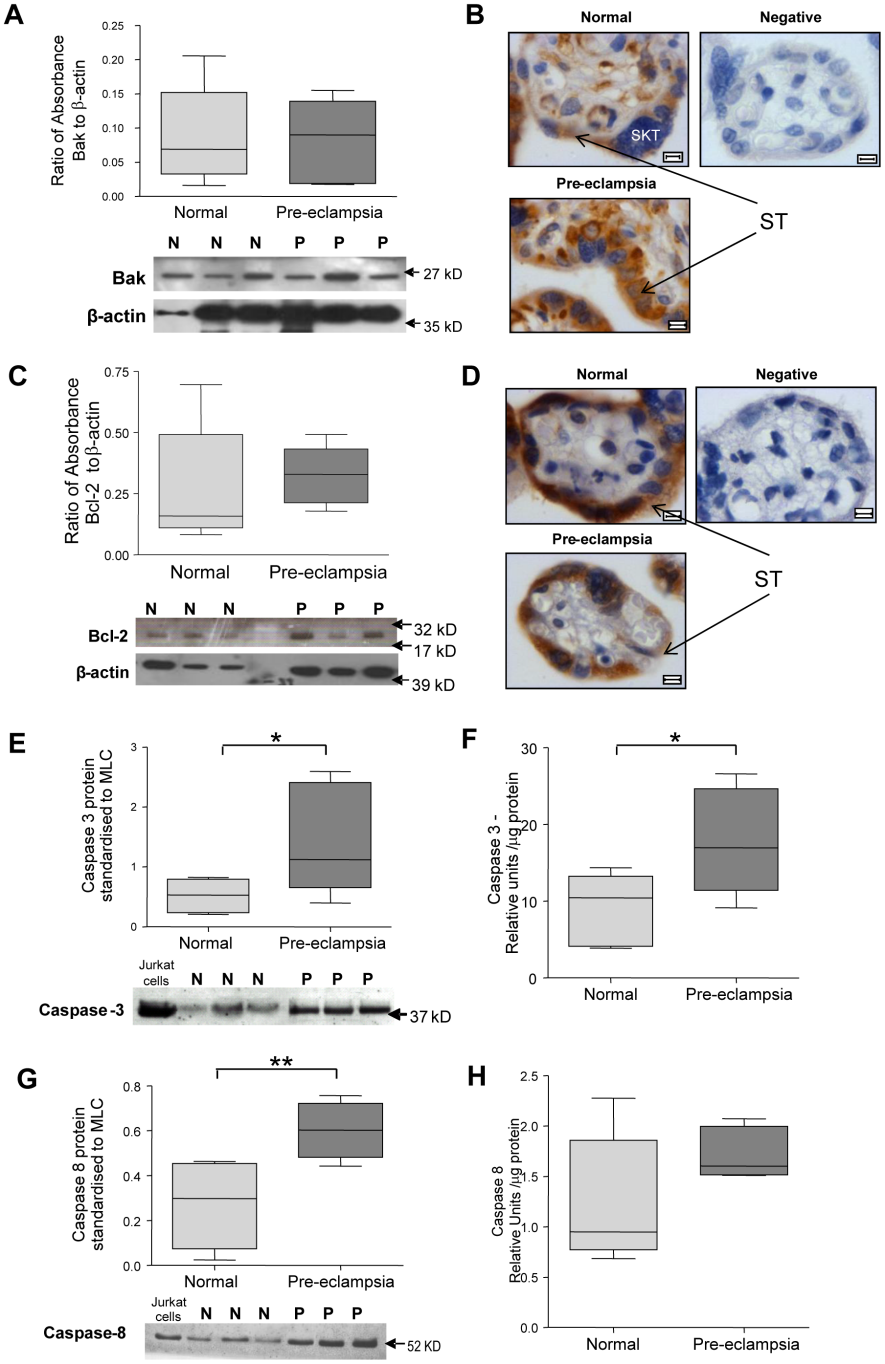
Placental expression of Bak, Bcl-2 and caspases 3 and 8 in normal pregnancy and pre-eclampsia (PE) at delivery. (**A, C, E and G**) densitometry for Bak, Bcl-2, caspase 3 and caspase 8, respectively, standardized to β-actin with representative blots included. (**B and D**) Localization of Bak and Bcl-2 respectively, in placental villi. ST =  Syncytiotrophoblast. Images counterstained with haematoxylin. Scale bar  = 5 µm. (**F and H**) mRNA expression of caspases 3 and 8 respectively in placental lysates (*p<0.05, **p<0.01, n = 8).

The expression of Bak and Bcl-2 in placental tissues showed no differences between study groups (Figure 2A and 2C). Bak localized to the cytoplasm of stromal cells, syncytiotrophoblast and cytotrophoblasts (Figure 2B). Unlike Bak, Bcl-2 was not observed in the cytoplasm of stromal cells and was only weakly expressed in cytotrophoblasts (Figure 2D).

Pro-caspase 3 expression was increased at the protein level in PE (Figure 2E), this increase was also evident in Caspase 3 activity (Figures 2E and F). Caspase 8 protein expression was significantly increased in PE (Figure 2G), but there was no statistically significant increase in activity (Figure 2H).

### Reducing Mdm2 increases p53 and alters trophoblast turnover

**Figure 3:**
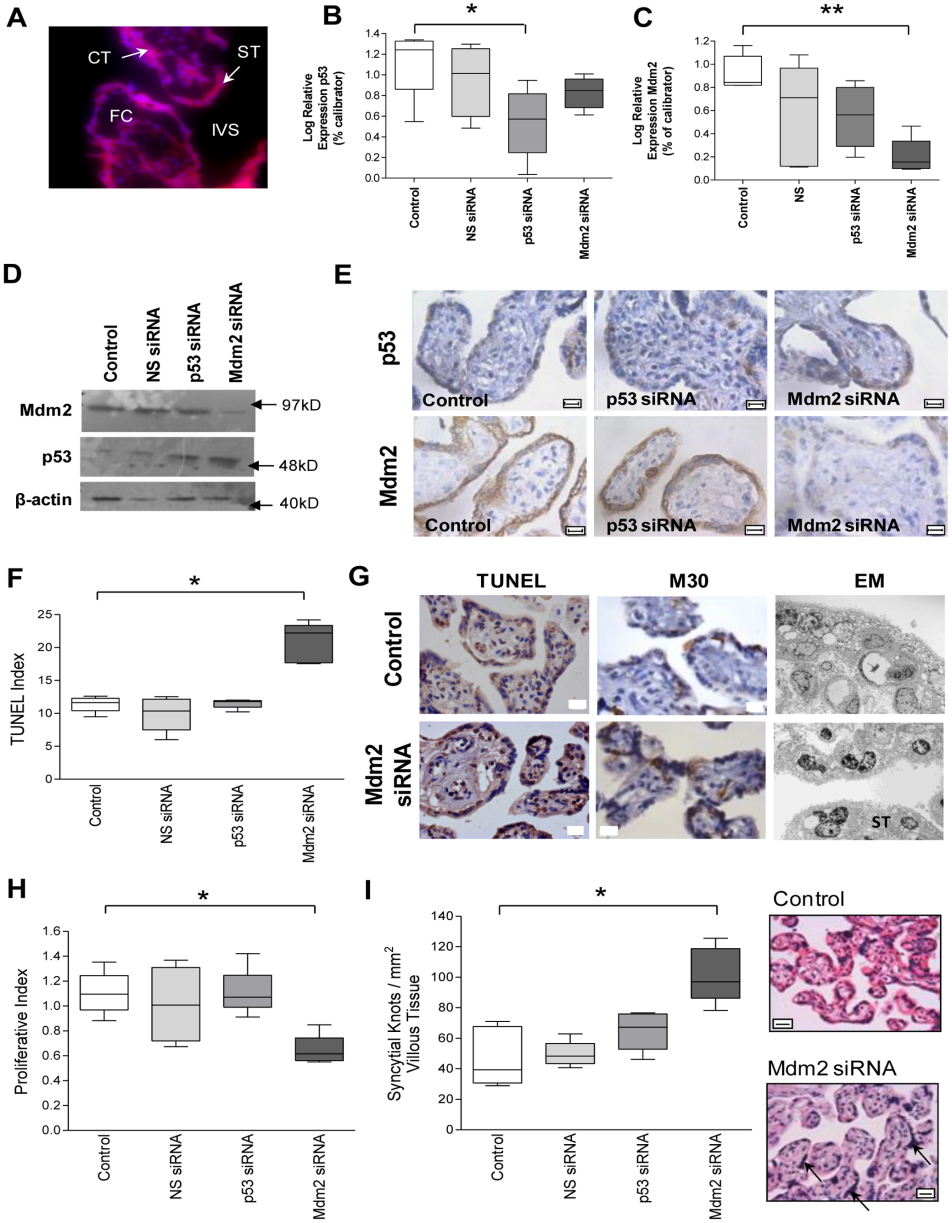
The apoptotic consequences of p53 and Mdm2 siRNA knockdown in human placental villous explants. (**A**) Transfection of placental explants with fluorescent labelled non-silencing siRNA demonstrating the presence of siRNA in cytotrophoblast (CT) and syncytiotrophoblast (ST). (**B and C**) Quantitative PCR demonstrating significant reductions in p53 and Mdm2 respectively, in explants treated with p53 or Mdm2 siRNA (*p< 0.05, **p<0.01, n = 6). (**D and E**) Western blot densitometry and immunohistochemistry showing a reduction in Mdm2 protein following treatment with Mdm2 siRNA. p53 expression is increased in trophoblast cytoplasm and nuclei in response to Mdm2 siRNA. (**F and G**) TUNEL index and images respectively, showing apoptosis significantly increased in explants exposed to Mdm2 siRNA (*p<0.05). Elevated apoptosis confirmed by M30 immunostaining with examples imaged by electron microscopy. (**H**) Proliferative index and representative images showing significantly reduced proliferation in explants cultured with Mdm2 siRNA (*p<0.05). (**I**) density of syncytial nuclear aggregates (SNA) increased by treatment Mdm2 siRNA (*p<0.05), representative images of control and Mdm2 siRNA (arrow  = SNA). Representative images shown (FC = fetal capillary, IVS = intervillous space, CT = cytotrophoblast, ST = syncytiotrophoblast). All scale bars  = 10 µm.

Following culture of term placental villous explants, transfection of the trophoblast layer with fluorescent labeled siRNA was evident (Figure 3A). Although p53 siRNA alone reduced the expression of p53 mRNA (Figure 3B), there was no reduction of p53 expression at the protein level (Figure 3D and E). Transfection with Mdm2 siRNA resulted in a decrease in Mdm2 expression (Figure 3C). This led to a decrease in Mdm2 protein and an increase in p53 protein expression which localized to the trophoblast layer (Figure 3D and E). There was no effect of scrambled siRNA or p53 siRNA on the expression of Mdm2.

The imbalance between p53 and Mdm2 in the Mdm2 silenced explants was associated with alterations in trophoblast turnover. The rate of apoptosis, shown by TUNEL staining, was significantly increased in the presence of Mdm2 siRNA (Figure 3F and G) as confirmed by M30 immunostaining (Figure 3G) and electron microscopy. The latter highlighting increased chromatin condensation and pyknosis in the trophoblast layer, following Mdm2 siRNA treatment in contrast to the euchromatic nuclei seen under control conditions (Figure 3G). Trophoblast proliferation measured by Mib-1 immunostaining was decreased in explants cultured in the presence of Mdm2 siRNA (Figure 3H). SNA density was increased in the presence of Mdm2 siRNA (Figure 3I). Scrambled siRNA failed to alter any aspect of cell turnover studied.

### p53 manipulation with Nutlin-3 and Pifithrin-α in BeWo cells

**Figure 4:**
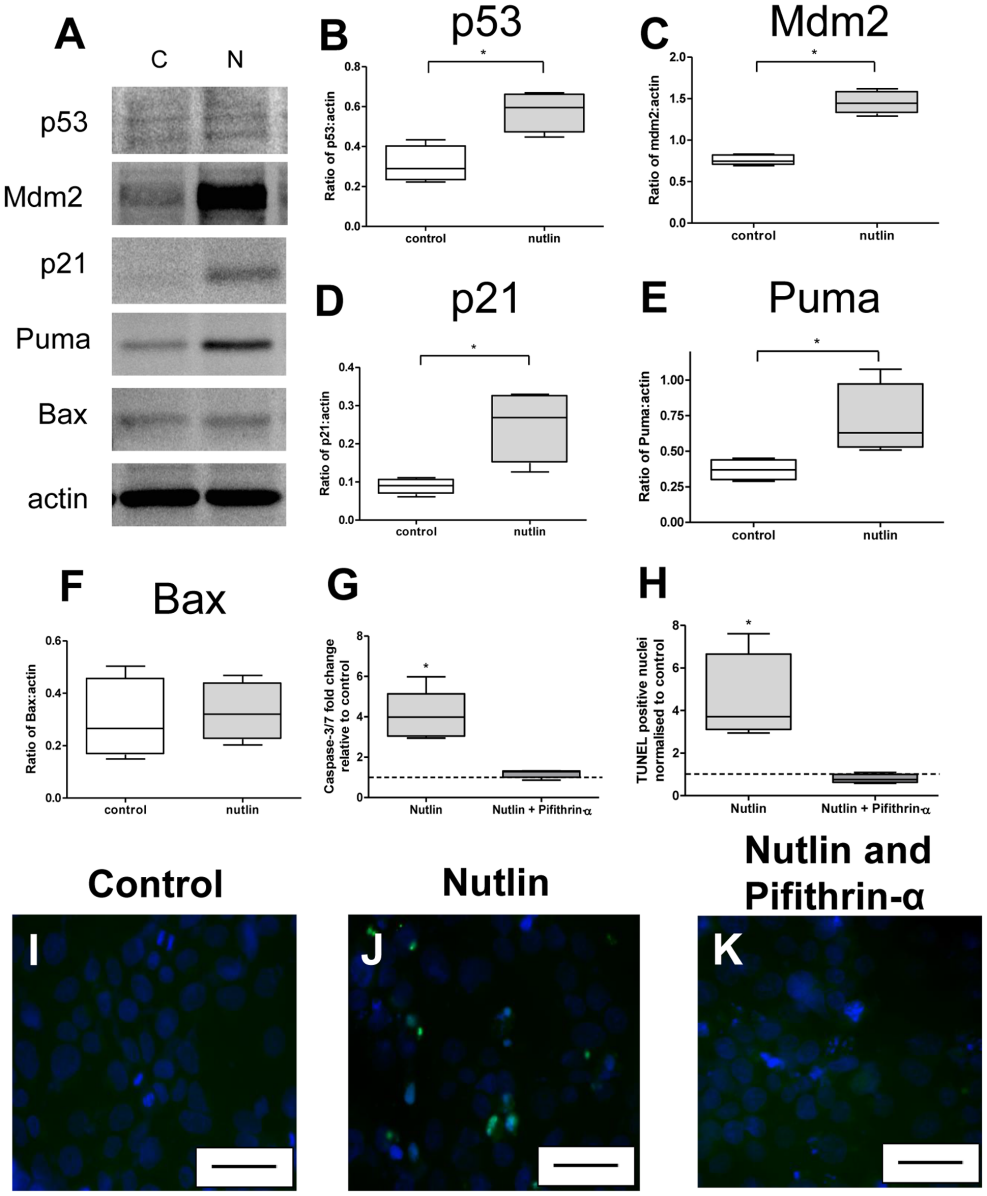
The modulation of protein targets of the p53 pathway by Nutlin-3 and Pifithrin-α in placental tissue. (**A**) Representative Western Blots of control and Nutlin-3 treated normal placental lysates. Densitometry revealed a significant increase in the expression of (**B**) p53, (**C**) Mdm2, (**D**) p21, (**E**) Puma (*p<0.05, n = 5). (**F**) There was no effect on Bax. Co-treatment of BeWo cells with Nutlin-3 (30 µM) and Pifithrin-α (10 µM) reduced caspase-3/7 activity (**G**) and TUNEL staining (**H**) to the level of controls. Representative images of TUNEL staining in (**I**) control, (**J**) Nutlin-3 and (**K**) co-treatment with Nutlin-3 and Pifithrin-α. Blue = DAPI, Green = TUNEL. Scale bar  = 50 µm.

**Figure 5:**
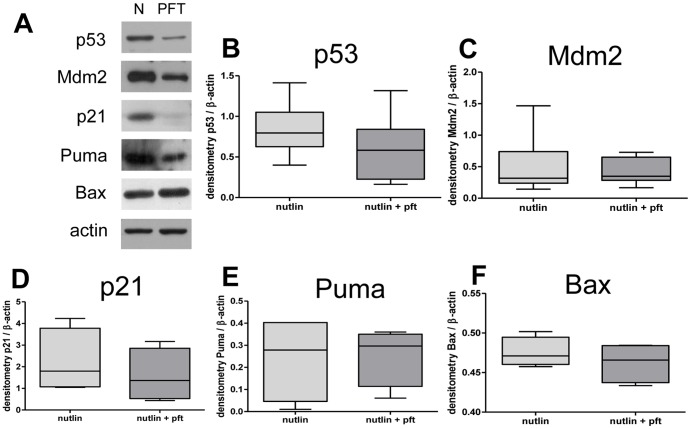
The modulation of protein targets of the p53 pathway by Nutlin-3 and Pifithrin-α in BeWo cells. (**A**) Western Blots of Nutlin-3 treated BeWo cell lysates and co-treatment with Nutlin-3 (30 µM) and Pifithrin-α (10 µM) demonstrated no effect upon (**B**) p53, (**C**) Mdm2, (**D**) p21, (**E**) Puma or (**F**) Bax protein expression (n = 5).

BeWo cells exposed to 30 µM Nutlin-3 for 24 hours showed an increase in p53, Mdm2, p21 and Puma at the protein level (Figure 4A–E), but no effect on Bax (Figure 4A and F). Treatment with Nutlin-3 increased apoptosis within BeWo cells as assessed by Caspase 3/7 activity. However, co-treatment with 10 µM Pifithrin-α abolished this effect (Figure 4G). A similar increase in TUNEL staining was observed in response to Nutlin-3 and attenuation with Pifithrin-α (Figure 4H–K). Western blot of BeWo lysates, following co-treatment with Nutlin-3 and Pifithrin-α, showed no change in p53, Mdm2, p21, Puma or Bax (Figure 5A–F).

### Effect of Nutlin-3 and Pifithrin-α on placental villous explants

**Figure 6:**
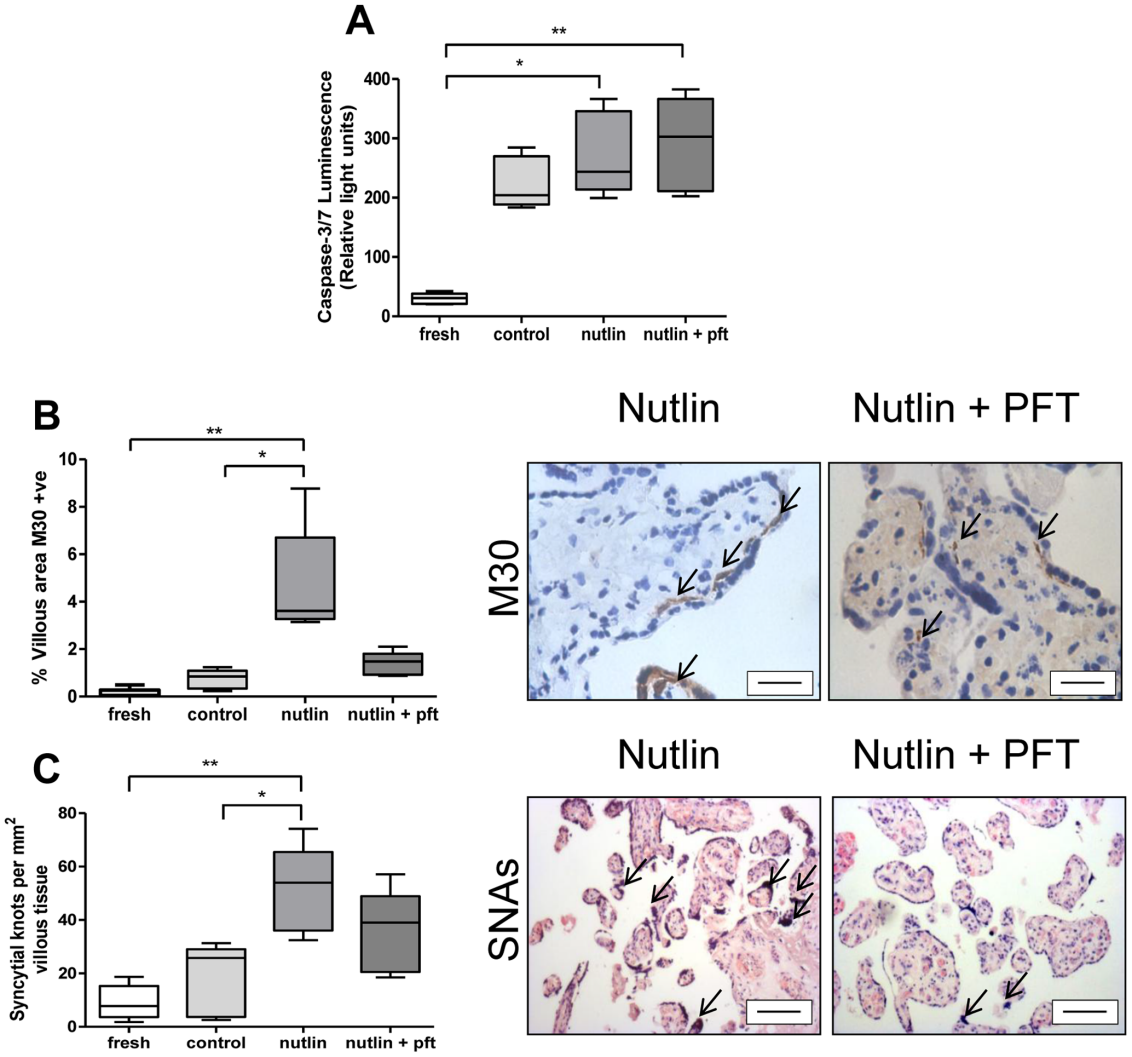
The modulation of p53 by Nutlin-3 and Pifithrin-α in placental villous explants. (**A**) Caspase-3/7 activity was increased by Nutlin-3 (30 µM), but unaffected by co-treatment with Pifithrin-α (10 µM). (**B**) Apoptosis assessed by M30 staining, highlighted by arrows, was increased by treatment with Nutlin-3 alone and reduced by co-treatment with Pifithrin-α (*p<0.05, **p<0.01, n = 5), (**C**) SNA density was increased by Nutlin-3 and reduced by Pifithrin-α, (SNAs marked by open arrows). Scale bar  = 50 µm.

**Figure 7:**
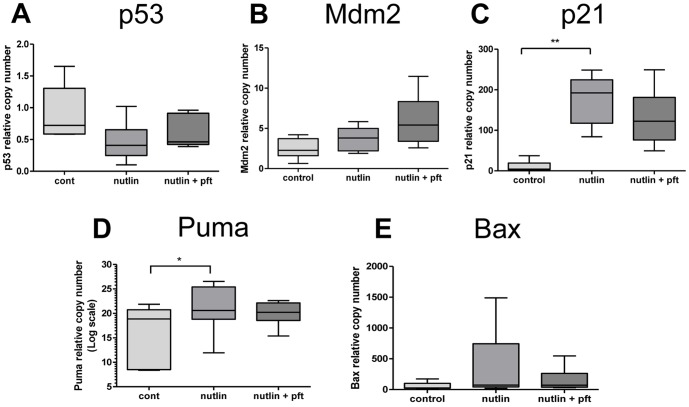
The expression of mRNA p53 targets in placental explants cultured with Nutlin-3 and Pifithrin-α. Co-treatment with Nutlin-3 (30 µM) and Pifithrin-α (10 µM) had no effect on (**A**) p53, (**B**) Mdm2 or (**E**) Bax mRNA expression. Treatment with Nutlin-3 increased (**C**) p21 and (**D**) Puma expression an effect lost by co-treatment with Pifithrin-α (*p<0.05, **p<0.01, n = 5).

Caspase-3/7 activity in homogenised explant tissue was increased following treatment with Nutlin-3; this increase was not reduced by Pifithrin-α co-treatment (Figure 6A). Treatment with Nutlin-3 increased M30 staining (Figure 6B) an effect reduced by co-treatment with Pifithrin-α. SNAs were increased by treatment with Nutlin-3, and notably this increase was reduced by Pifithrin-α co-treatment, in a response similar to M30-recognition (Figure 6C). Treatment with Nutlin-3 and Pifithrin-α had no impact on mRNA expression in placental explants for p53, Mdm2 or Bax (Figure 7A, B and E). A significant increase in p21 and Puma mRNA was observed with Nutlin-3 treatment, but here again this was reduced by concomitant Pifithrin-α treatment (Figure 7C and D).

## Discussion

This study has demonstrated changes in the protein expression of p53 and downstream components of this pathway in villous trophoblast in PE. Furthermore, we have shown that disruption of the balance between pro-apoptotic p53 and anti-apoptotic Mdm2 results in exaggerated apoptosis in culture models of villous trophoblast. These observations are consistent with other reports of increased p53 and Bax in invasive extravillous trophoblast and villous trophoblast in PE and FGR, where apoptosis is particularly associated with sites of trophoblast damage [22], [33], [54], [55], [56].

Our data regarding p53, Mdm2, Bax, Bak and Bcl-2 localization is in accordance with other reports of term placental tissue [13], [57], [58], [59], [60]. The majority of proteins regulating apoptosis are localized to the villous trophoblast compartment, with little or no expression in the stroma. The increase in caspase 3 related to increased staining for cytokeratin-M30 indicating increased caspase 3 activity in trophoblast [50]. Under normal circumstances, the syncytiotrophoblast of pregnancy is protected against apoptosis, maintaining viability and continued mRNA transcription and protein translation [61]. The integrity of this crucial syncytium is sustained by the expression of several anti-apoptotic proteins: Mdm2, Bcl-2, Mcl-1 and inhibitors of apoptosis proteins (IAPs) which antagonize the effects of pro-apoptotic p53, Bax/Bak, Mtd and smac [14], [21], [62]. In contrast, we have noted a significant reduction in the anti-apoptotic protection of the syncytiotrophoblast in PE, with decreased Mdm2 facilitating an increase in p53 expression, creating a pro-apoptotic environment, evident from downstream p21 and exaggerated Bax. The expression of proteins such as Bak and Bcl-2, the transcription of which is not promoted by p53, were not altered in PE, confirming that the constituents of the intrinsic apoptotic pathway were not only present in villous trophoblast in this study, but also that downstream effects of p53 are similar to those described in other systems [26], [63].

Evidence of placental dysfunction in PE has implicated endoplasmic reticulum (ER) stress, unfolded protein response (UPR), increased autophagy and apoptosis [3], [4], [5]. These pathways are not discrete with shared components and regulators [64], [65]. For p53, cellular localization can alter the balance between autophagy and apoptosis [66], but ultimately several mechanisms could explain the stimulation of p53 in PE, including hypoxia and oxidative stress [67], [68]. Given current thinking, increased p53 could facilitate initial survival of oxidatively stressed placental tissues, secondary to ischaemia-reperfusion injury. In this scenario, elevated p53 would initially promote the transcription of p21, a cell cycle inhibitor, to induce cell-cycle arrest and autophagy, but particularly severe or prolonged oxidative stress could eventually encourage apoptosis, through an imbalance in pro- and anti-apoptotic proteins, such as p53, Mdm2, Bax and Bcl-2 or Mtd and Mcl-1 [21]. Thus, p53 may be involved at several levels of the cellular dysfunction seen in PE, playing a role in the exaggerated autophagy and apoptosis in response to tissue damage.

In PE, the increased expression of p53 was present in the trophoblast layer as were the downstream transcription-dependent effects of p21 and Bax. Historically, the syncytiotrophoblast was thought to be transcriptionally inactive although recently this has been challenged, at least in normal tissues [69], particularly in the syncytiotrophoblast of the first trimester placenta [61]. Our data supports the hypothesis that the syncytiotrophoblast retains its capacity for RNA transcription.

An imbalance between p53 and Mdm2 expression, and their proposed role in the regulation of apoptosis in PE, was confirmed in placental explants, whereby the loss of function of Mdm2 was associated with exaggerated apoptosis and syncytial degeneration. Following treatment with siRNA, the pronounced decrease in Mdm2 protein *in vitro*, as compared to p53, may be explained by (i) differences in protein longevity, with stabilized p53 having a half-life of 24 hours compared to approximately one hour for Mdm2 [70], [71], or by (ii) a preferential reduction in cytoplasmic (Mdm2) over nuclear protein (p53) [72]. Nevertheless, this increased accumulation of p53, in the absence of Mdm2, is consistent with evidence from gene knockout studies, in which the relationship between mdm2 and p53 has been shown to be essential for normal placental development [32].

To further confirm the key importance of p53 for trophoblast survival and function, we used the highly selective p53-activator, Nutlin-3 [38]. The concentrations used were comparable with published data [73], [74], but higher than others [38], [75], suggesting variations in sensitivity between cell types and tissues. In this case discrepancies may reflect the inherent anti-apoptotic profile of syncytialised trophoblast, as previously reported *in vitro* [76]. In addition to increased apoptosis, BeWo cells exposed to Nutlin-3 showed increased p53, Mdm2, p21 and Puma expression. This suggests that p53 exerted some of its effects via a nuclear transcriptional route producing a similar pattern of protein expression to other cell types: an increase in p53 [38], [77], [78], [79], [80], Mdm2 [77], [79], [80], p21 [38], [77], [79], [80] and Puma [77], [78]. However, there was no direct effect on Bax, a feature also previously observed [77].

The use of the p53-inhibitor, Pifithrin-α, reduced Nutlin-3-induced apoptosis in a way similar to that observed in other cells [40], [81], [82]. These results differ from an alternative study on primary trophoblast, in which Pifithrin-α increased trophoblast apoptosis, as measured by cleaved cytokeratin-18, co-treated with Nutlin-3 [75]. This may reflect differences in cell type between BeWo and primary trophoblast. The lack of a noticeable effect upon p53, Mdm2, p21, Puma or Bax protein expression in BeWo cells, co-treated with Pifithrin-α, complicates an appreciation of its inhibitory effect, particularly as the terminal steps of apoptosis were prevented. However, possible explanations, as defined in other studies are: (i) alterations in p53 and/or Mdm2 activity or interactions, rather than protein levels *per se* [83], (ii) caspase-8 dependent mechanisms which negate cytoplasmic migration of p53 to the nucleus [84], or (iii) increased trafficking of Fas receptor from the Golgi to the cell surface which may occur following p53 activation [85]. Pifithrin-α has been shown to reduce caspase-8 and Fas-induced apoptosis in myoblasts [86] and epithelial cells [81], exposed to Doxorubicin. Interestingly a similar pattern with Nutlin-3 and Pifithrin-α was observed in placental explants suggesting that (i) the BeWo model is appropriate for trophoblast, and (ii) Nutlin-3 is a potent inducer of p53 mediated apoptosis within both BeWo cells and placental explants, and (iii) Pifithrin-α is less effective at reversing p53-induced apoptosis in whole tissue.

In summary, these experiments suggest that dysregulation of the p53 pathway seen in late-onset PE is capable of inducing apoptosis in human trophoblast. Further work would be needed to verify such as change in early-onset PE (<28 weeks) and the full relationship between severity of maternal disease, placental apoptosis and expression of p53. However, with confirmation of conserved interactions with p53 and downstream cell cycle regulators in trophoblast, along with susceptibility to pharmacologic modulation, p53 may conceivably hold potential as a therapeutic target for improvements in placental-related disease and pregnancy outcome.
